# Quantitative assessment of renal functions using ^68^Ga-EDTA dynamic PET imaging in renal injury in mice of different origins

**DOI:** 10.3389/fmed.2023.1143473

**Published:** 2023-03-27

**Authors:** Ying Ding, Yu Liu, Li Zhang, Yinqian Deng, Huanyu Chen, Xiaoli Lan, Dawei Jiang, Wei Cao

**Affiliations:** ^1^Department of Nuclear Medicine, Union Hospital, Tongji Medical College, Huazhong University of Science and Technology, Wuhan, China; ^2^Hubei Key Laboratory of Molecular Imaging, Wuhan, China; ^3^Key Laboratory of Biological Targeted Therapy, The Ministry of Education, Wuhan, China

**Keywords:** nuclear medicine, glomerular filtration rate, PET imaging, kidney injury, ^68^Ga-EDTA

## Abstract

**Background:**

Early detection of kidney diseases can be challenging as conventional methods such as blood tests or imaging techniques (computed tomography (CT), magnetic resonance imaging (MRI), or ultrasonography) may be insufficient to assess renal function. A single-photon emission CT (SPECT) renal scan provides a means of measuring glomerular filtration rates (GFRs), but its diagnostic accuracy is limited due to its planar imaging modality and semi-quantification property. In this study, we aimed to improve the accuracy of GFR measurement by preparing a positron emission tonometry (PET) tracer ^68^Ga-Ethylenediaminetetraacetic acid (^68^Ga-EDTA) and comprehensively evaluating its performance in healthy mice and murine models of renal dysfunction.

**Methods:**

Dynamic PET scans were performed in healthy C57BL/6 mice and in models of renal injury, including acute kidney injury (AKI) and unilateral ureter obstruction (UUO) using ^68^Ga-EDTA. In a 30-min dynamic scan, PET images and time-activity curves (TACs) were acquired. Renal function and GFR values were measured using renograms and validated through serum renal function parameters, biodistribution results, and pathological staining.

**Results:**

^68^Ga-EDTA dynamic PET imaging quantitatively captured the tracer elimination process. The calculated GFR values were 0.25 ± 0.02 ml/min in healthy mice, 0.01 ± 0.00 ml/min in AKI mice, and 0.25 ± 0.04, 0.29 ± 0.03 and 0.24 ± 0.01 ml/min in UUO mice, respectively. Furthermore, ^68^Ga-EDTA dynamic PET imaging and GFR_PET_ were able to differentiate mild renal impairment before serum parameters indicated any changes.

**Conclusions:**

Our findings demonstrate that ^68^Ga-EDTA dynamic PET provides a reliable and precise means of evaluating renal function in two murine models of renal injury. These results hold promise for the widespread clinical application of ^68^Ga-EDTA dynamic PET in the near future.

## 1. Introduction

The assessment of renal function is crucial in determining patient treatment and management in clinical practice. The most commonly used clinical measurements include serum creatinine (Cr), urea nitrogen (BUN), and cystatin C (Cys-C) to rapidly and cost-effectively reflect renal function (1). As the glomerular filtration rate (GFR) decreases, these biomarkers increase, particularly when GFR falls below 60 ml/min (1.73 m^2^) (2). However, due to the wide range and frequent fluctuations of these serum markers, it can be difficult to detect increased serum levels and respond to renal injury in a timely manner. Measured GFR (mGFR) provides a quantitative evaluation of renal filtration capacity, but the gold standard for measuring GFR, which involves testing plasma and urinary clearance rates of inulin, is not widely used due to its high cost and its time-consuming and repetitive natures (3). Imaging-based techniques to measure renal function have been developed and may replace traditional methods, which include iohexol-enhanced computed tomography (CT) (4) and gadolinium-enhanced magnetic resonance renography (MRR) (5, 6), as well as nuclear medicine renal imaging. These techniques can directly reflect changes in renal anatomy and visualize the passage of contrast agents through the kidney. However, the use of milligram-scale CT or magnetic resonance (MR) contrast agents can cause tubular reabsorption (7), and large doses may increase the risk of allergy or nephrotoxicity (8).

Nuclear imaging techniques, such as single-photon emission CT (SPECT) and positron emission tomography (PET), provide dynamic renal imaging by using traces of radioactive probes that are specifically filtered or reabsorbed by the kidney to visualize excretion and measure GFR. The most commonly used nuclear imaging method to assess renal function in clinical practice is the ^99m^Tc-diethylenetriaminepentaacetic acid (^99m^Tc-DTPA) renal scan, which plays a crucial role in the assessment of unilateral renal function, renovascular hypertension, hydronephrosis, and renal transplantation. However, GFR values obtained from ^99m^Tc-DTPA SPECT imaging can often be unreliable, especially in patients with renal abnormalities and excessive body weight or in pediatric patients. This is due to the limitations of SPECT imaging, including non-individualized renal depth correction and semi-quantitative capabilities, which may result in biased GFR values (9–11). Furthermore, SPECT planar imaging provides limited information, especially in patients with space-occupying lesions in the kidney, leading to inaccurate and redundant examinations and increased costs.

Positron emission tonometry presents a possible solution to assess GFR in a non-invasive manner. The superior three-dimensional (3D) capabilities of PET detectors improve quantitative and localization accuracy compared to the limitations of SPECT planar imaging. This allows for the direct measurement of GFR by integrating information related to the spatial position of the kidney and the excretion kinetics of the tracer. Additionally, the high sensitivity of PET imaging enables the use of a lower administration activity compared to SPECT, reducing the risk of toxic effects and radiation exposure to ensure patient safety (12). This feature makes PET a particularly advantageous option for pediatric patients and those with severely impaired renal function, where an accurate assessment of GFR is crucial in making informed clinical treatment decisions.

Ethylenediaminetetraacetic acid (EDTA) is a well-known chelating agent and has a history of use in radiochemistry research. It has a robust capacity to form stable complexes with most metal ions and is known to be entirely filtered by the renal glomerulus (13, 14). ^51^Cr-EDTA has properties similar to inulin, and its blood clearance rate is extensively used to estimate GFR in Europe. However, due to the limited availability of Cr-51, this method is gradually being phased out. Compared to DTPA, EDTA has significantly less binding to plasma proteins, leading to rapid and specific glomerular filtration (15). Ga-68, with its short half-life (68 min) and high decay energy, obtained from a ^68^Ge-^68^Ga generator, is an excellent choice for renal PET imaging. These properties make ^68^Ga-EDTA a promising candidate to meet the requirements of an ideal renal tracer. Our previous studies have confirmed that the renal excretion curve of ^68^Ga-EDTA aligns well with a one-compartment elimination pharmacokinetic model curve (16). Dynamic imaging data from ^68^Ga-EDTA PET/CT can be used to directly calculate total GFR and split renal GFR in mice. In this study, we aimed to comprehensively evaluate the potential application of ^68^Ga-EDTA in the quantification of GFR in healthy animals as well as in mice with renal dysfunction, such as rhabdomyolysis-induced acute kidney injury (AKI) and unilateral ureter obstruction (UUO), and to provide a user-friendly GFR measurement procedure.

## 2. Materials and methods

### 2.1. Materials

All chemicals were purchased from Aladdin Biochemical Technology Co., Ltd. (China) as reagent grade and used as received without further purification unless otherwise stated.

### 2.2. Radiolabeling and quality control

Ga-68 was eluted from a ^68^Ge/^68^Ga-generator (ITG, Germany) with the eluent of 0.05 M HCl. ^68^Ga-EDTA was prepared by mixing 10 μl of EDTA (0.5 M, pH = 8.0) and 37 MBq of ^68^GaCl_3_ (17, 18), and pH was adjusted to ~5 by adding the NaOAc buffer (0.02 M, pH = 6.8). Radiochemical purity (RCP) of ^68^Ga-EDTA was performed using instant thin-layer chromatography (TLC) silica gel plates (Agilent Technologies, Inc., CA, USA), developed with 0.2 M sodium acetate (pH = 4.5), and analyzed by a TLC scanner (Zhongcheng Tech. Co., Ltd., Hefei, China). Radiolabeling efficiency was determined by the γ-counter (2470 Automatic Gamma Counter, WIZARD, PerkinElmer, Norwalk CT, USA) (*n* = 3). The stability of ^68^Ga-EDTA *in vitro* was examined in phosphate buffer solution (PBS) and fetal bovine serum (FBS). ^68^Ga-EDTA was mixed with PBS and 10% FBS in a ratio of 1:2 and incubated in a water bath at 37°C. The mixture supernatant was developed using ITLC-SG and analyzed by TLC and the γ-counter at 30, 60, and 120 min.

### 2.3. Animal studies

Animal experiments were performed on male C57BL/6 mice (8 weeks, 19–22 g, Hubei Biont Biotechnology Co., Ltd., China). All animal protocols were reviewed and approved by the Institutional Animal Care and Use Committee of Tongji Medical College of Huazhong University of Science and Technology.

#### 2.3.1. Establishment of the AKI model

All mice were deprived of water for 24 h before the establishment of AKI. Glycerol (50% v/v in 0.9% saline) was injected into the hind limb of each mouse (8 ml/kg, *i.m*.) after water deprivation. PET imaging and biodistribution studies were performed 24 h after injection.

#### 2.3.2. Establishment of the UUO model

All mice were anesthetized with 2% isoflurane (RWD Life Science Co., Ltd., Shenzhen, China). A vertical incision was made in the abdominal wall of the mouse, exposing the left ureter and the lower pole of the kidney. The ureter was ligated in two points using a 4.0 silk and cut in half. The wound was sewn up using a 4.0 silk. Operations were performed in a sterile environment to avoid animal infection. Scanning experiments were performed on postoperative days 1, 8, and 22.

### 2.4. Small animal dynamic PET/CT renal imaging

^68^Ga-EDTA dynamic PET was performed on Trans PET Discoverist 180 (RAYCAN Technology Co., Ltd., Suzhou, China). Mice were anesthetized with 2% isoflurane and then placed on a scanner bed with their tail vein catheterized. Along with the 30-min dynamic PET scan, simultaneous intravenous injection of ^68^Ga-EDTA (2.80 ± 0.24 MBq/50 μl) was performed. Based on our previous study, tracer injection was accomplished within the initial 10 s to obtain an eligible blood perfusion curve in the kidneys. CT scanning was performed directly after PET scanning and reconstructed with an image matrix size of 512^*^512^*^512. The dynamic image was reconstructed using the 3-D ordered subset expectation maximization algorithm with point spread function modeling (OSEM-3D-PSF) reconstruction algorithm. The 30-min list-mode files were divided into 28 frames (10 s^*^6, 30 s^*^6, 60 s^*^6, and 120 s^*^10). For each frame, we implemented an OSEM-3D-PSF with two iterations and 12 subsets. The image matrix size for OSEM-3D-PSF is 320 × 320 × 200 with isotropic voxel sizes of 0.5 mm × 0.5 mm × 0.5 mm.

### 2.5. Renogram analysis

The reconstructed PET/CT data were processed and analyzed using Siemens Inveon Research Workplace software version 4.2 for volume-of-interest (VOI) analysis. CT images and PET/CT fusion images were used to guide the VOIs due to the partial volume effect. We manually delineated the VOIs of the heart, bilateral kidneys, and bladder. The Inveon Research Workspace was used to automatically calculate the radioactivity within the VOIs of each frame. We located standardized VOIs of the heart's left ventricle near the cardiac apex with a volume of 27 voxels (3^*^3 ^*^3) to reduce the influence of cardiac muscles. The VOIs of the kidneys were delineated by the CT images, especially for animals with a renal disease. We defined elliptical VOIs in the bilateral kidneys that are appressed to the lower pole of the kidney and semiautomatically adjusted them according to the radioactive distribution in the kidney. We defined VOIs of the bladder by their signal intensity on CT images. Time-activity curves (TACs) were generated using the radioactivity in each frame in VOIs plotted against the time point of each frame. The renogram was defined as TAC of each kidney. In addition, for further quantitative assessment, four novel indicators were used. Time-to-peak (*T*_max_), peak value, and time to half-maximum (*T*_1/2_) were obtained by analyzing the renogram (19). Blood perfusion parameter (BP) was denoted as the slope of the renogram from 0 min to *T*_max_, and renal elimination parameter (EP) was denoted as the slope of the renogram from *T*_max_ to 30 min.

### 2.6. GFR calculation

The area under the curve (AUC) method was used to calculate the mouse GFR (20–23). Our previous study demonstrated the reliability of this method compared with others and showed good consistency with the blood clearance of ^68^Ga-EDTA (16). Briefly, we hypothesized that ^68^Ga-EDTA was excreted by the kidney at a stable rate, and this radiopharmaceutical was only filtered through glomerulus irreversibility; the GFR of each mouse can be calculated as:

(1) $$\begin{array}{l} \text{GFR }=\;\frac{\text{Activit}{\text{y}}_{\text{bladder}}\left(30\;\min\right)}{\int_{0\;\min}^{30\;\min}\text{Activit}{\text{y}}_{\text{plasma}}\text{dt}}\; \end{array}$$

The radioactivity in plasma was corrected with the blood radioactivity concentration and hematocrit (Hct), as mentioned in the following formula (24, 25):

(2) $$\begin{array}{l} \text{Activit}{\text{y}}_{\text{plasma}}\left(t\right)=\text{Activit}{\text{y}}_{\text{blood}}\left(t\right)\times \frac{1}{\left(1-Hct\right)}\; \end{array}$$

Referring to Gate's method, we defined the split GFR of each kidney as the ratio of AUC_splitkidney_ to AUC_bilateralkidneys_ from 1 to 2 min multiplied by the total GFR, and the formula is:

(3) $$\begin{array}{l} \text{Split GFR}=\frac{{\text{AUC of split kidney}}_{1-2\;\min}}{{\text{AUC of bilateral kidneys }}_{1-2\;\min}}\times \text{GFR } \end{array}$$

### 2.7. Biodistribution study

The blood, brain, heart, lung, liver, spleen, kidneys, stomach, small intestine, large intestine, muscle, bone, and whole tail were taken 30 min after the tracer injection. Samples were washed three times with PBS, dried, and weighed. The radioactivity of each sample was determined using a γ-counter. The results of tissues' uptake were decay-corrected.

### 2.8. Biological toxicity tests and histopathology

Blood and tissue samples were collected from mice 24-h post-injection. The blood samples were collected for routine blood tests, Cr, BUN, serum alanine aminotransferase (ALT), and aspartate aminotransferase (AST). Tissues were collected after blood collection and fixed with 4% paraformaldehyde at room temperature for 24 h for hematoxylin and eosin (H&E) and periodic acid-Schiff (PAS) staining.

### 2.9. Statistical analysis

GraphPad Prism software (Version 7.0, GraphPad Software, Inc., USA) and Origin 2021 software (OriginLab, Northampton, UK) were used to produce figures and compute statistical analyses. The quantitative data are presented as the mean ± standard deviation (SD). A one-way analysis of variance (ANOVA) test was performed to compare the difference between the healthy mice and the renal dysfunctional mice. The results were statistically significant when the *p*-value was <0.05.

## 3. Results

### 3.1. Radiochemistry

A facile radiolabeling method was developed by mixing ^68^Ga (eluted with 0.1 M HCl) and EDTA (0.5 M, pH 8.0) for 10 min in a sodium acetate buffer (0.2 M, pH ~6.8). The labeling efficiency of ^68^Ga-EDTA was over 99%. The stability of ^68^Ga-EDTA was evaluated in PBS and FBS for 2 h and showed no significant decomposition (~95%), as shown in Supplementary Figure S1. These results indicate that ^68^Ga-EDTA is a stable complex suitable for further investigation.

### 3.2. ^68^Ga-EDTA dynamic PET/CT imaging of healthy mice

**Figure 1:**
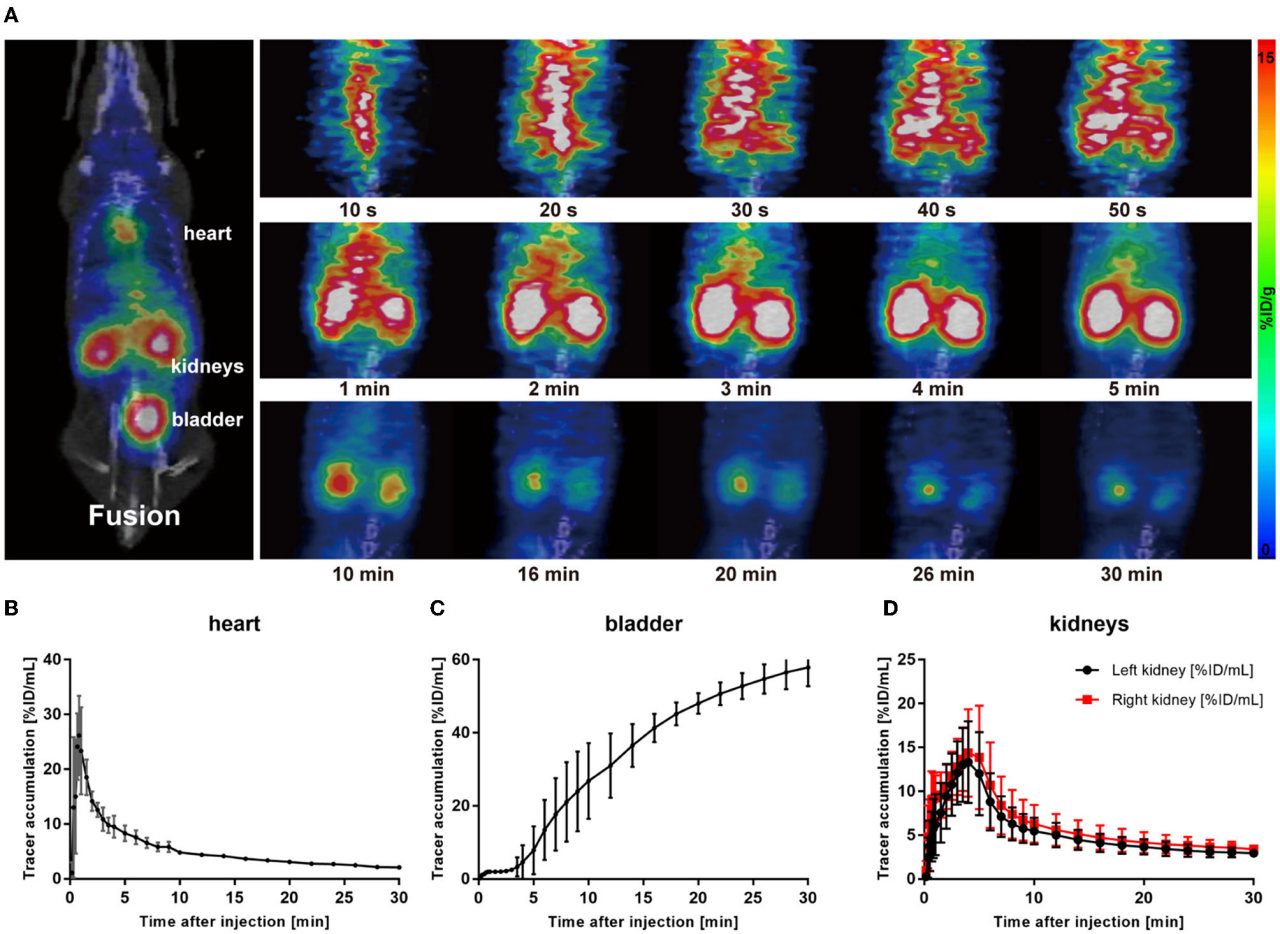
^68^Ga-EDTA dynamic positron emission tonometry (PET)/computed tomography (CT) imaging of healthy mice. **(A)** Coronal fusion images of ^68^Ga-EDTA dynamic PET/CT scanning in healthy mice after intravenous injection of ^68^Ga-EDTA. **(B)** The heart time-activity curve (TAC) of healthy mice was obtained *via* PET imaging. **(C)** The bladder TAC of healthy mice was obtained *via* PET imaging. **(D)** The renograms of healthy mice were obtained *via* PET imaging.

To demonstrate the performance of ^68^Ga-EDTA eliminated in healthy mice, a 30-min dynamic PET scan was performed (Figure 1A). ^68^Ga-EDTA was injected as a bolus through a tail vein catheter; this tracer circulated within the blood promptly within the first 10 s after injection and reached the bilateral kidneys as early as 40 s p.i., and the contour of the kidneys can be immediately observed. Up to 50 s p.i., ^68^Ga-EDTA basically flowed out from the heart, and the kidneys were delineated ever more clearly. The renal cortex and pelvis can be distinguished from 1 to 6 min. At ~5 min, a small amount of tracers started to appear in the bladder. This rapid renal elimination of ^68^Ga-EDTA confirmed that the tracer was primarily filtered by the renal glomerulus, revealing negligible potential renal tubular reabsorption and secretion. No other obvious tracer retention can be seen in images during scanning, indicating that extra-renal elimination of ^68^Ga-EDTA can be largely excluded. Biodistribution experiments were carried out at 30 min p.i. (**Table 3** and **Figure 6**). Remaining ^68^Ga-EDTA in the blood and organs were low. In the end of observation (30 min), the tracer had mainly accumulated in the bladder, and the blood concentration was lower than 0.3%ID/ml. Rapid renal excretion and low organ accumulation indicate the high biocompatibility of ^68^Ga-EDTA. In the toxicity test, the routine blood test, renal function, and liver function parameters showed that ^68^Ga-EDTA had no effects on renal function, hematopoietic function, or hepatic function (Supplementary Table S1). Pathological analysis of the tissue sections did not show any negative effects of ^68^Ga-EDTA on the major organs (Supplementary Figure S3).

Radioactivity counts gained from four volumes of interest (VOIs) were plotted against time and then used to form a TAC of ^68^Ga-EDTA (Figures 1B–D). Renogram specially refers to TAC of the split kidney (Figure 1D). The renal function was quantified by parameters mentioned in the method, including the peak value, *T*_max_, *T*_1/2_, BP, and EP (Table 1). Kidneys of healthy C57BL/6 mice have a rich supply of blood and powerful filtration capacity. Once the tracer entered the kidneys, it perfused with circulation and achieved dynamic balance during rapid distribution within the initial 60 s. During this process, the tracer was continuously filtered by the renal glomerulus. When the filtration was greater than the perfusion, the renogram started to present a downward trend. The excretion curve of ^68^Ga-EDTA was fitted using a two-exponential decay (Supplementary Figure S2) with a *T*_1/2 − Fast_ of 1.93 ± 0.59 min and a *T*_1/2 − Slow_ of 17.54 ± 7.92 min. The tracer in the kidneys raised to the peak (*T*_max_) at 3.81 ± 0.96 min with an average peak value of 14.78 ± 3.89%ID/ml. The blood perfusion in healthy kidneys was defined as BP with a ratio of 3.57 ±0.76%ID/ml·min for the right kidney and 3.95 ± 0.73%ID/ml·min for the left kidney. After arriving at the peak, the tracer started rapid diffusion followed by elimination. The kidneys excreted half of the tracer (*T*_1/2_) at 7.63 ± 1.51 min. The average elimination speed of healthy mice was denoted as EP with a ratio of −0.48 ± 0.19%ID/ml·min for the right kidney and −0.43 ± 0.14%ID/ml·min for the left kidney. The GFR of healthy C57BL/6 mice calculated by ^68^Ga-EDTA dynamic PET imaging is 0.25 ± 0.02 ml/min (Table 2), and the split GFR for the right and left kidney is 0.14 and 0.11 ml/min, respectively.

**Table 1:** Renogram information.

		**Normal**	**AKI**	**UUO-1d**	**UUO-8d**	**UUO-22d**
Peak value (%ID/mL)		14.78 ± 3.89	4.82 ± 0.56^*^	13.58 ± 6.20	12.00 ± 2.25	11.62 ± 0.69
T_max_ (min)		3.81 ± 0.96	3.13 ± 1.30	4.38 ± 3.84	2.17 ± 0.29	2.50 ± 0.87
T_1/2_ (min)		7.63 ± 1.51	Not reached	14.00 ± 4.00	10.00 ± 6.93	7.67 ± 2.52
BP	R	3.57 ± 0.76	2.45 ± 1.41^*^	3.78 ± 1.48	5.52 ± 0.36	5.11 ± 2.00
(%ID/mL·min)	L	3.95 ± 0.73	1.39 ± 0.53^*^	1.54 ± 0.86^*^	0.77 ± 0.10^*^	0.55 ± 0.01^*^
EP	R	−0.48 ± 0.19	−0.05 ± 0.01	−0.39 ± 0.27	−0.31 ± 0.06	−0.32 ± 0.03
(%ID/mL·min)	L	−0.43 ± 0.14	−0.04 ± 0.01	0.42 ± 0.32	0.17 ± 0.12	0.04 ± 0.01

All parameters were compared with those of healthy mice individually using the one-way analysis of variance (ANOVA) test.

* p < 0.05.

**Table 2:** ^68^Ga-EDTA dynamic positron emission tonometry- (PET-) derived glomerular filtration rate (GFR).

		**Normal**	**AKI**	**UUO-1d**	**UUO-8d**	**UUO-22d**
Total GFR (mL/min)		0.25 ± 0.02	0.01 ± 0.00^***^	0.25 ± 0.04	0.29 ± 0.03	0.24 ± 0.01
AUC_1 − 2min_ (%)	R	0.55	0.57	0.68	0.80	0.78
	L	0.44	0.43	0.32	0.20	0.22
Split GFR (mL/min)	R	0.14	0.007	0.17	0.24	0.19
	L	0.11	0.005	0.08	0.06	0.05

All parameters were compared with those of healthy mice individually using the one-way ANOVA test.

*** p < 0.001.

### 3.3. ^68^Ga-EDTA dynamic PET/CT imaging of AKI mice

Acute kidney injury is a serious but common clinical syndrome which is induced by drug toxicity, renal parenchymal disease, or renovascular disease (26). Glycerol-induced AKI is one of the most widely used models of acute renal dysfunction. The high dose of glycerol injected in the muscle induces rhabdomyolysis and results in a decrease in renal blood perfusion and renal tubular necrosis. In this study, we used the glycerol-AKI model to assess whether ^68^Ga-EDTA PET scanning was suitable for the diagnosis of renal dysfunction. Within 24 h of glycerol injection, nephron necrosis was obviously observed in renal tissue sections, the renal tubular lumen became swollen, and renal tubular epithelial cells started desquamation and necrosis (**Figure 5**).

**Figure 2:**
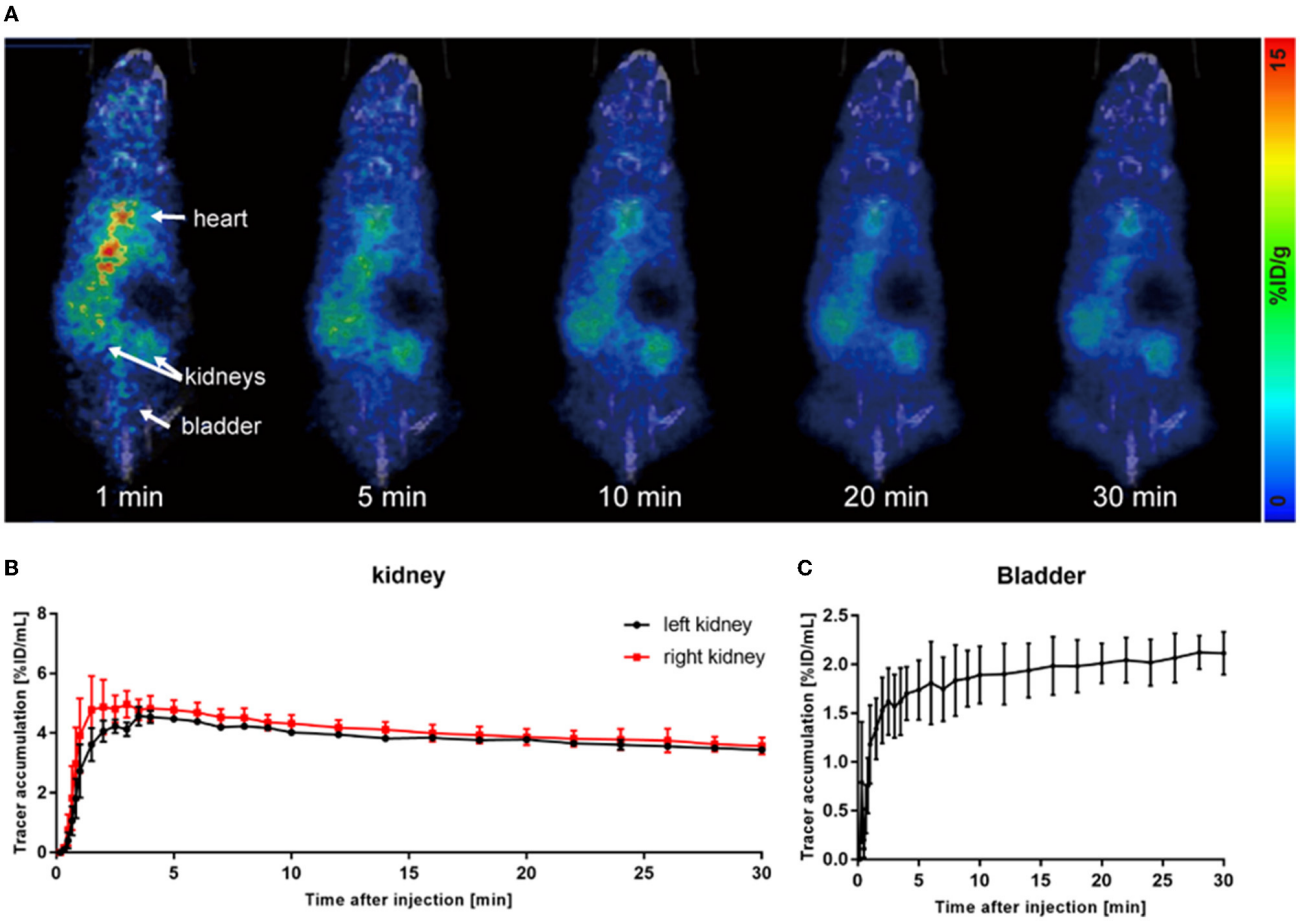
^68^Ga-EDTA dynamic PET/CT imaging of acute kidney injury (AKI) mice. **(A)** Coronal fusion images of ^68^Ga-EDTA dynamic PET/CT scanning in AKI mice. **(B)** The renograms of AKI mice were obtained *via* PET imaging. **(C)** The bladder TAC of AKI mice was obtained *via* PET imaging.

As shown in Figure 2A, most of the tracers circulated in the blood pool after injection, and only a small amount of tracers entered the kidneys. Eventually, almost no tracer was observed to be excreted into the bladder (Figure 2C). Due to the damage to the excretory capacity, the tracer still remained at a relatively high level in the heart until the end of observation. Renograms of AKI kidneys showed a low and flat curve (Figure 2B).

The quantitative analysis of the AKI renograms (**Figures 4G**, **H**) demonstrated that the kidneys of rhabdomyolysis-induced AKI had a reduced blood supply and impaired excretory function. ^68^Ga-EDTA reached the peak at 3.13 ± 1.30 min (*T*_max_) in kidneys of AKI, with an average peak value of 4.82 ± 0.56%ID/ml, and the average BP for the right kidney was 2.45 ± 1.41 and 1.39 ± 0.53%ID/ml·min for the left. The EP was −0.05 ± 0.01 and −0.04 ± 0.01%ID/ml·min for the right and left kidneys, respectively. All the indicators were lower than healthy (*p* < 0.05). No discernible downward trend appeared in the renograms over the observation period, and less than half of the tracer was excreted.

The average GFR value of AKI mice was 0.01 ± 0.00 ml/min, which was significantly lower than that of healthy mice (**Figure 4C**). The split GFR for right and left kidneys was 0.007 and 0.005 ml/min, respectively. For a more intuitive contrast, we assessed Cr and BUN in mice, both of which were significantly higher than in healthy mice (**Figures 4A**, **B**). Radioactivity biodistribution experiments were performed to verify the accuracy of dynamic PET scanning (Table 3 and **Figures 6B**, **C**). At 30 min p.i., the radioactivity in the blood exceeds 2.8%ID/g, which is 10 times higher than normal. In addition, all organs had a higher accumulation of radioactivity than healthy organs, which is consistent with the results from the PET images.

**Table 3:** Biodistribution of ^68^Ga-EDTA in five groups.

**Tissue**	**Control (%ID/g)**	**AKI (%ID/g)**	**UUO-1d (%ID/g)**	**UUO-8d (%ID/g)**	**UUO-22d (%ID/g)**
Blood	0.266 ± 0.023	2.812 ± 2.463	1.338 ± 0.411	1.770 ± 0.631	1.223 ± 0.568
Brain	0.014 ± 0.005	0.107 ± 0.064	0.133 ± 0.086	0.152 ± 0.041	0.068 ± 0.046
Heart	0.056 ± 0.014	0.476 ± 0.519	0.328 ± 0.098	0.353 ± 0.124	0.237 ± 0.174
Lung	0.131 ± 0.039	1.479 ± 1.231	0.881 ± 0.318	1.232 ± 0.416	0.789 ± 0.496
Liver	0.205 ± 0.110	1.284 ± 0.267	1.034 ± 0.200	1.434 ± 0.919	0.708 ± 0.325
Spleen	0.708 ± 0.040	2.822 ± 1.438	3.465 ± 1.172	2.808 ± 1.735	1.252 ± 0.529
Kidneys	1.072 ± 0.230	7.416 ± 3.968	20.422 ± 5.582	8.088 ± 1.933	3.043 ± 0.804
Stomach	0.115 ± 0.096	0.755 ± 0.918	0.467 ± 0.099	0.521 ± 0.145	0.318 ± 0.211
Small intestine	0.052 ± 0.020	0.301 ± 0.268	0.206 ± 0.063	0.288 ± 0.046	0.137 ± 0.089
Large intestine	0.060 ± 0.024	0.500 ± 0.475	0.485 ± 0.409	0.474 ± 0.200	0.159 ± 0.090
Muscle	0.031 ± 0.010	0.431 ± 0.347	0.524 ± 0.396	0.462 ± 0.174	0.238 ± 0.152
Bone	0.163 ± 0.088	0.957 ± 0.804	0.919 ± 0.601	0.747 ± 0.380	0.325 ± 0.228

### 3.4. ^68^Ga-EDTA dynamic PET/CT imaging of UUO mice

**Figure 3:**
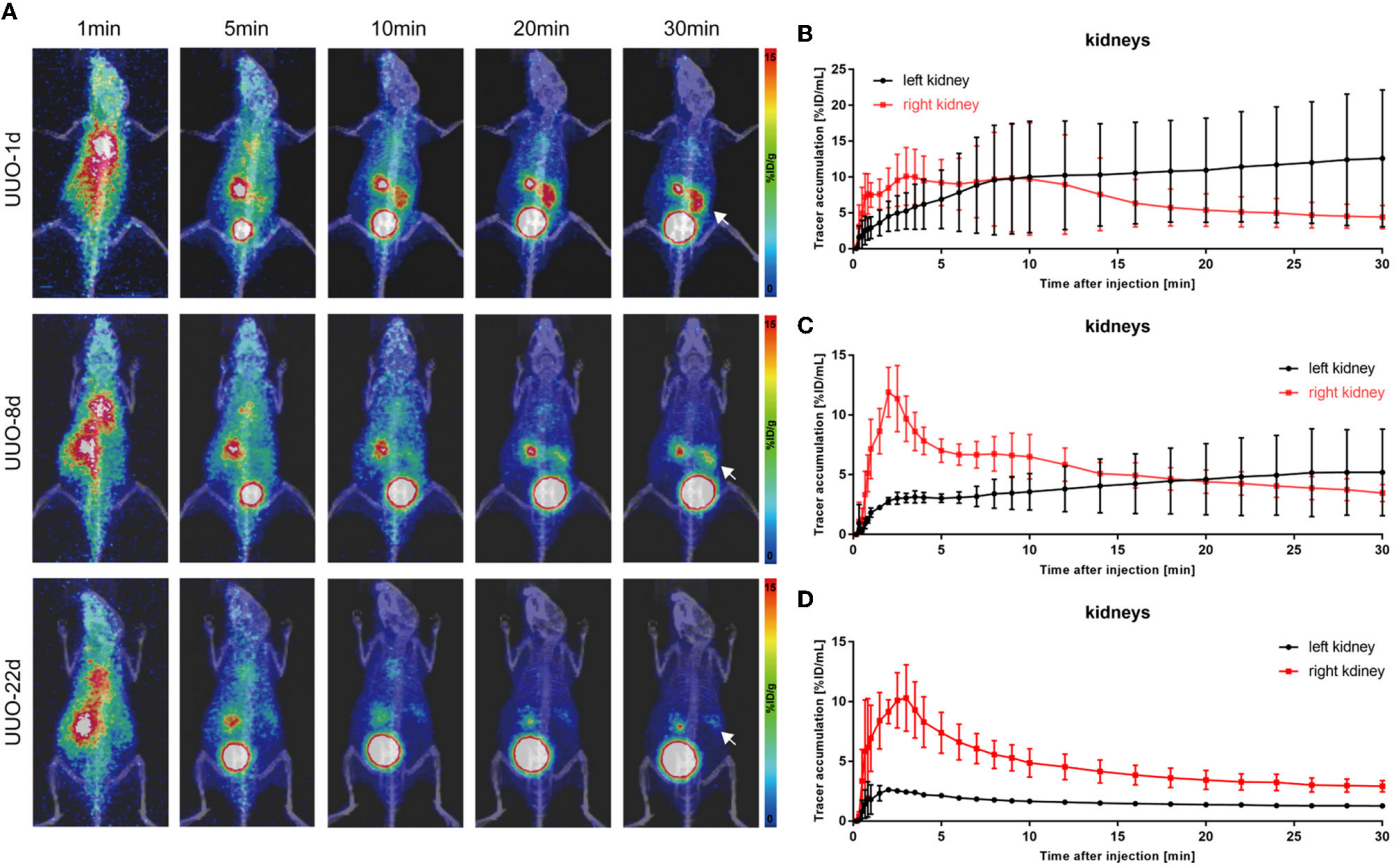
^68^Ga-EDTA dynamic PET/CT imaging of unilateral ureter obstruction (UUO) in mice and the vital renograms. **(A)** Coronal fusion images of ^68^Ga-EDTA dynamic PET/CT scanning on UUO mice on days 1, 8, and 22 after UUO (white arrows, the left ureter was ligated). **(B)** The renograms of UUO-1d mice were obtained *via* PET imaging. **(C)** The renograms of UUO-8d mice were obtained *via* PET imaging. **(D)** The renograms of UUO-22d mice were obtained *via* PET imaging.

Unilateral ureter obstruction is a typical model of chronic renal tubulointerstitial fibrosis. This model was established by extensive ligation of one of the ureters, and the destruction of the renal parenchyma in the obstructed kidney continued over time, posing a challenge for the sustainable dynamic observation of renal function. PET scanning was performed on UUO mice on post-operative days 1, 8, and 22. As shown in Figure 3, ^68^Ga-EDTA dynamic PET images on different days showed functional changes in the split kidneys. The performance of the bilateral kidneys in ^68^Ga-EDTA dynamic PET scanning is completely different after ureteral ligation. ^68^Ga-EDTA can be excreted through the counter-lateral kidney and flows normally into the bladder, but it can also be found stuck in the obstructed side. Furthermore, we found the radioactivity contour of the obstructed kidney gradually fading away.

**Figure 4:**
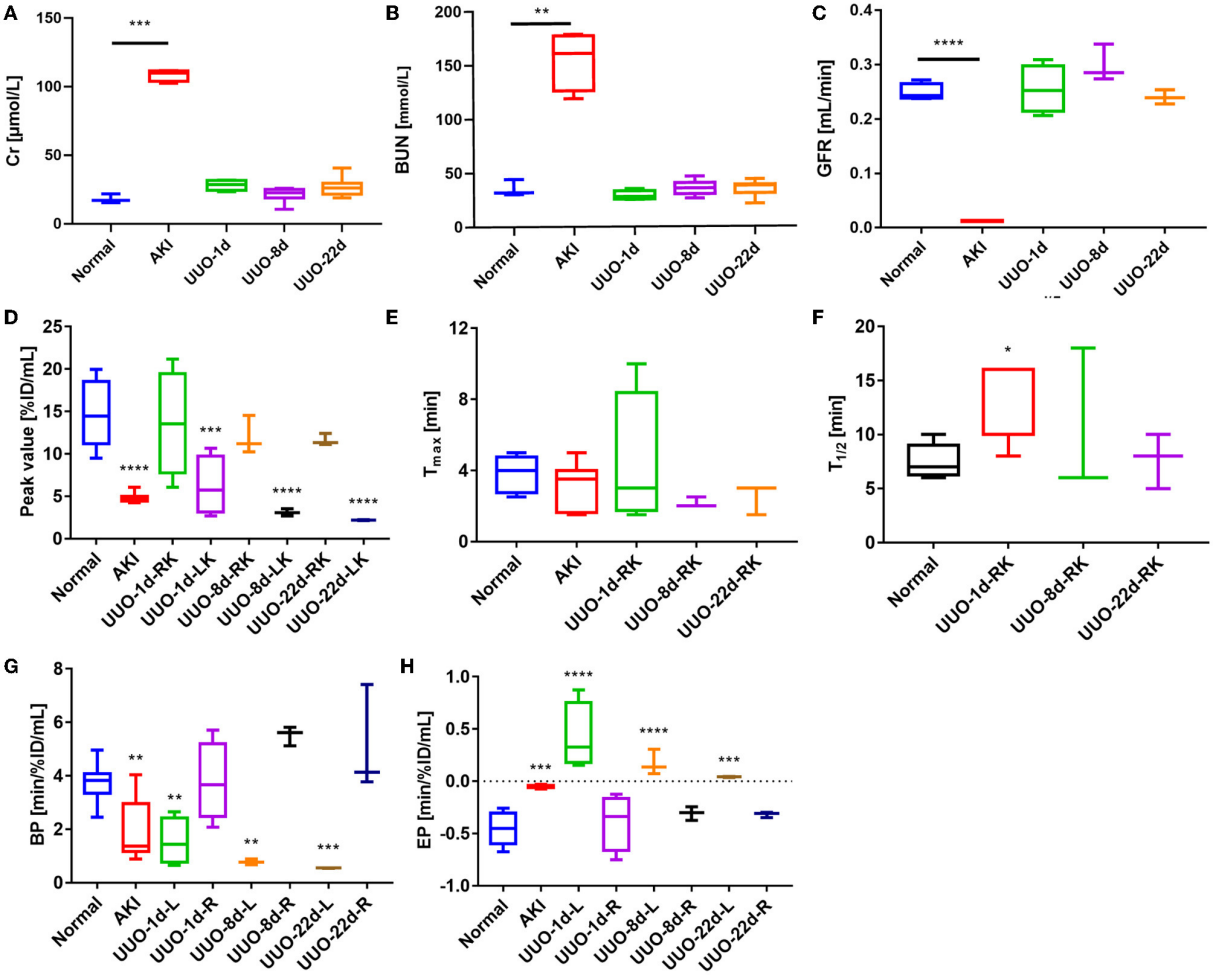
Renal function of healthy mice and mice with renal dysfunction, as represented by glomerular filtration rate (GFR), creatinine (Cr), urea nitrogen (BUN), and other figure parameters. **(A)** The Cr values of healthy mice and mice with renal dysfunction. **(B)** BUN values of healthy mice and mice with renal dysfunction. **(C)** The GFR values of healthy mice and mice with renal dysfunction. **(D)** The renogram peak value of healthy mice, AKI mice, and the split kidneys in UUO mice. *The peak value of the obstructed kidney (left kidney) in UUO mice was 4 min p.i. **(E)** The renogram *T*_max_ value of healthy mice, AKI mice, and the right kidney in UUO mice. **(F)** The renogram *T*_1/2_ value of healthy mice, AKI mice, and the right kidney in UUO mice. **(G)** The renogram BP of healthy mice, AKI mice, and the split kidneys in UUO mice. **(H)** The renogram EP of healthy mice, AKI mice, and the split kidneys in UUO mice. *p < 0.05, **p < 0.01, ***p < 0.001, ****p < 0.0001.

The renograms of UUO mice (Figures 3B–D) and the quantified information (Figures 4D–H) guided us with further analysis (Figures 3B–D). The renogram of the obstructed kidney at 1 day after the operation was a continuously rising curve that reflected hydronephrosis, but this curve gradually decreased over time. To calculate the BP and EP for the obstructed kidney, we set the *T*_max_ as 4 min. The blood perfusion (BP) of the obstructed kidney was 1.54 ± 0.87%ID/ml·min on day 1, 0.77 ± 0.10%ID/ml·min on day 8, and 0.54 ± 0.01%ID/ml·min on day 22, which decreases over time (Figure 4G). Correspondingly, the elimination (EP) of the obstructed kidney was presented in full contrast to healthy, which is 0.42 ± 0.32%ID/ml·min on day 1, 0.17 ± 0.12%ID/ml·min on day 8, and 0.04 ± 0.01%ID/ml·min on day 22. The contralateral healthy kidney, as the only remaining excretory organ, maintains a highly stable filtration capacity (Figure 4H). The BP of the contralateral kidney was 3.78 ± 1.48%ID/ml·min on post-operative day 1, increasing to 5.52 ± 0.36%ID/ml·min on day 8 and 5.11 ± 2.00%ID/ml·min on day 22, which are equal to the healthy kidney or relatively higher. The elimination (EP) of the contralateral kidney remained stable over the course of the study, which was −0.39 ± 0.27%ID/ml·min on day 1, −0.31 ± 0.06%ID/ml·min on day 8, and −0.32 ± 0.03%ID/ml·min on day 22. In general, the image-quantified parameters of the contralateral kidney, such as *T*_max_, *T*_1/2_, BP, and EP, were basically the same as those of healthy mice with no statistical difference (Table 1). To our surprise, our calculated GFR values of UUO mice showed no statistical difference from those of healthy mice. The values of Cr and BUN also reflected this circumstance (Figures 4A, B). The average GFR value of UUO mice changed from 0.25 ± 0.04 ml/min on day 1 to 0.29 ± 0.03 ml/min on day 8 and then to 0.24 ± 0.01 ml/min on day 22. However, the GFR_PET_ showed better sensitivity in the evaluation of split renal function. Significant differences were found between the GFR values of the bilateral kidneys in UUO mice (Table 2). The GFR value of the compensatory kidneys in UUO mice was higher than that of the unilateral healthy kidney and reflects the physiologic adjustment of the mice.

**Figure 5:**
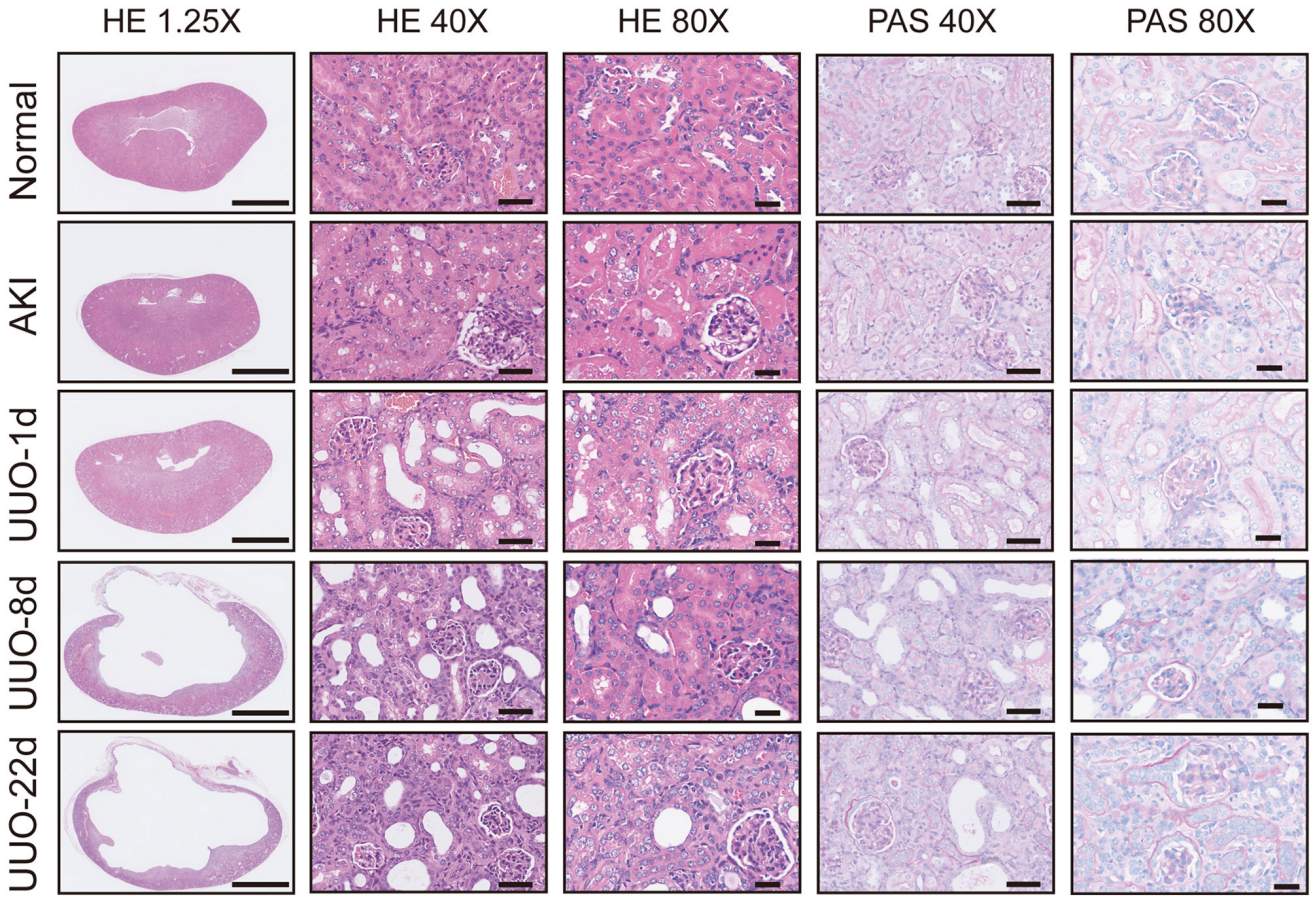
Pathological sections of healthy kidneys and kidneys with renal dysfunction. Scale bar for 1.25×: 2.5 mm, 40×: 50 μm, 80×: 25 μm.

**Figure 6:**
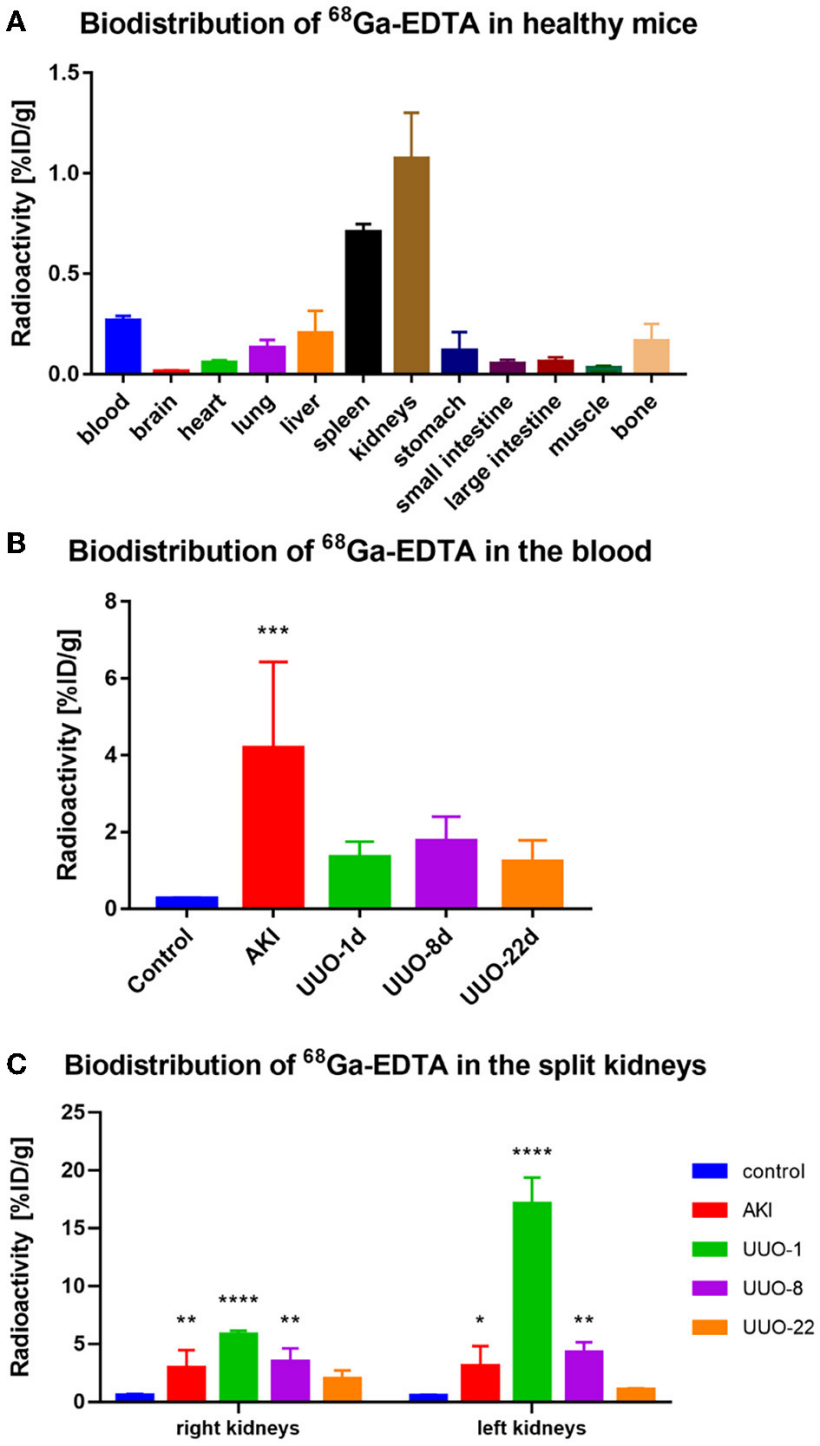
The biodistribution of ^68^Ga-EDTA *in vivo*. **(A)** The biodistribution of ^68^Ga-EDTA in healthy mice 30-min post-injection. **(B)** The biodistribution of ^68^Ga-EDTA in the blood in healthy mice and other mice with renal dysfunction 30 min post-injection. **(C)** The biodistribution of ^68^Ga-EDTA in split kidneys among the different groups. *p < 0.05, **p < 0.01, ***p < 0.001, ****p < 0.0001.

Hematoxylin and eosin and PAS section staining of the kidney revealed the pathological changes that occurred in the obstructed kidney, such as the presence of dilated cortical kidney tubules and medullar atrophy as well as the hydronephrosis and renal cortical compression (Figure 5). These changes are confirmed by PET images and the results of radioactivity biodistribution as well (Figure 6C).

### 3.5. Comprehensive evaluation of renal function

**Figure 7:**
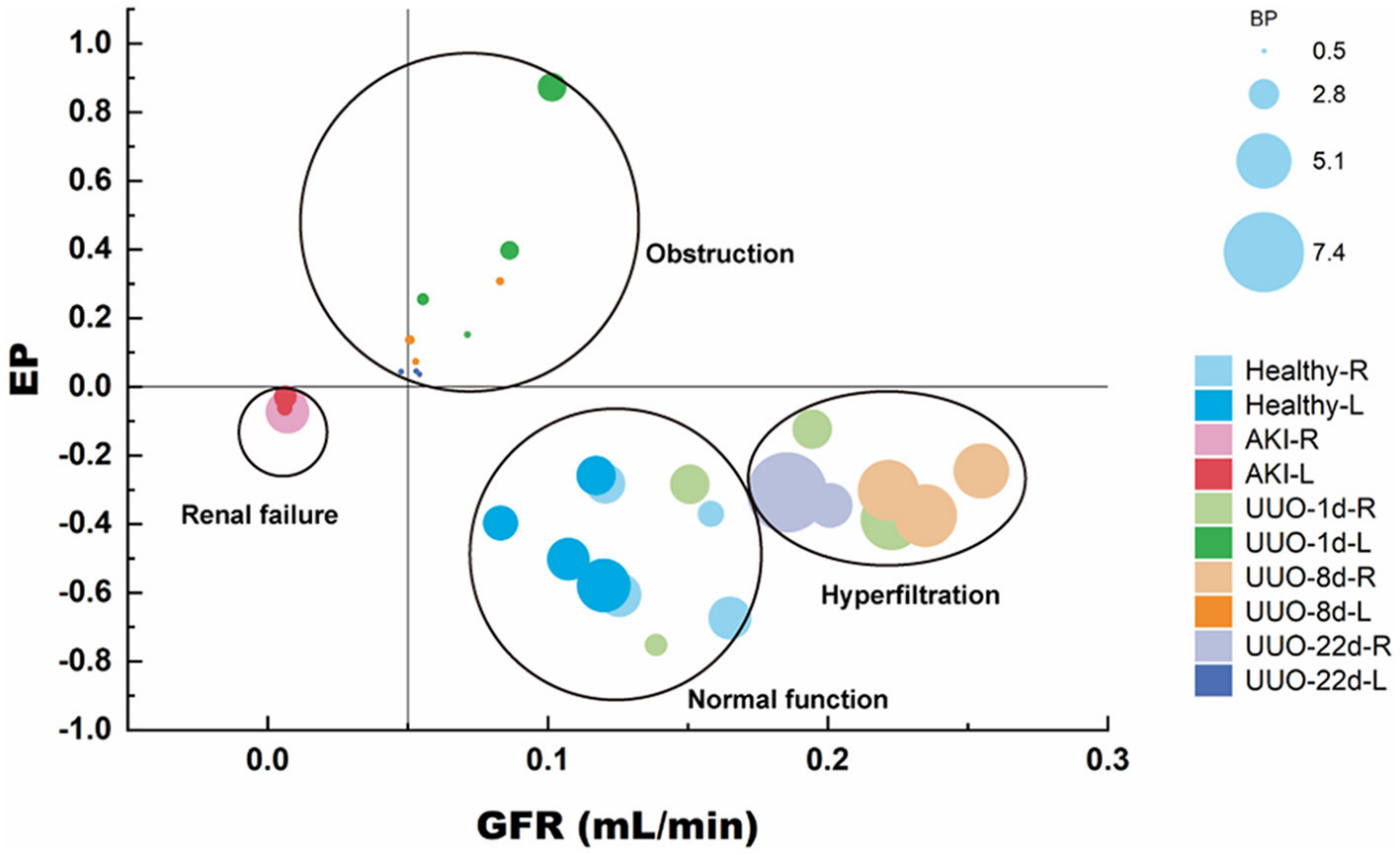
Renal function evaluation on synthetic data as reflected/estimated by GFR_PET_.

We used ^68^Ga-EDTA dynamic PET imaging and renograms to comprehensively evaluate mouse renal function. Figure 7 shows the advantage of PET renal scanning for measuring split renal function, and the bubble plot provides a comprehensive and integrated analysis of the split renal function and the overall condition of the examinees, as we accept that renal function should not be solely based on the GFR value. The bubble plot indicates that normal glomerular filtration requires an adequate blood supply and rapid efflux of the tracer through the urinary tract. Glycerol-induced AKI kidneys had a lower BP than healthy ones, and the obstructed renal tubules resulted in very weak excretion, which helps us classify AKI kidneys as renal failure. EP was positive for obstructed kidneys in UUO mice due to the obstruction in the urine outflow. Thus, we were able to identify the obstruction in the urinary tract. The contralateral kidney may have compensatory filtration, resulting in a higher BP and GFR than healthy ones. Therefore, we suggest that the evaluation of a particular renal function should involve multiple factors rather than GFR alone.

## 4. Discussion

In light of the obvious superiority of PET in image quality and quantification accuracy over SPECT, it was anticipated that more organ and tissue function evaluations will shift to the PET modality. Accurate quantification of renal function in terms of GFR, one of the territories where SPECT imaging used to dominate, has been challenged by its outdated two-dimensional (2D) images and loss of information during reconstruction. Several studies (16, 24, 25, 27–29) have reported the application of PET/CT in the evaluation of renal function and have demonstrated the advantages of PET/CT in the assessment of renal function, especially for those patients with complex urinary problems (14). Many tracers for renal PET imaging were proposed (10, 29). Our previous study demonstrated that ^68^Ga-EDTA might be an appropriate choice for the assessment of renal function, and the one-compartment pharmacokinetic method can be used for efficient quantification of GFR (16, 18). In this study, we comprehensively evaluated the performance of ^68^Ga-EDTA as a renal glomerular filtration tracer using healthy C57BL/6 mice. We further expand on the application of ^68^Ga-EDTA dynamic PET/CT scanning in animals with renal dysfunction, including AKI and UUO, especially investigating the dynamic functional changes in the course of renal dysfunction. We demonstrated the application potential and translational value of ^68^Ga-EDTA dynamic PET/CT scanning in the quantification of GFR.

Special needs were required for the measurement of renal function. The tracer for assessing GFR must be completely filtrated through the renal glomeruli and have no renal tubular reabsorption or secretion. Our study demonstrated the metabolism process of ^68^Ga-EDTA *in vivo* and *in vitro*. ^68^Ga-EDTA remained stable for 2 h. This complex can only be rapidly removed from the circulation *via* the kidneys but not cleaved. ^68^Ga-EDTA also has an extremely low binding to blood plasma protein (15), which makes it an excellent probe for the assessment of renal GFR. The washout curves of ^68^Ga-EDTA in the heart and kidneys of healthy mice fit well to the two-exponential decay model, but the washout curves from the injured kidneys in mice with renal dysfunction were not suitable for this model. A one-compartment pharmacokinetic model was stable enough for the vast majority of kidney elimination processes (30), and filtration efficiency can be calculated using the AUC method through PET imaging-derived data. In our study, the ^68^Ga-EDTA-derived GFR of healthy mice was 0.25 ± 0.02 ml/min, and the split GFR for right and left kidneys was 0.14 and 0.11 ml/min, respectively. This GFR value was highly consistent with our previous study and other studies (16, 31). In addition, the upgrade of the operation process increases the reproducibility and generalizability of the method.

Next, we validated the application of ^68^Ga-EDTA dynamic PET/CT scanning in mice with renal dysfunction and demonstrated that this method is preferable for assessing renal injury, especially at early stages. Consistent with physiological changes (such as decreased blood perfusion or sluggish excretion), PET imaging detected kidney filtration alterations and was more sensitive than Cr examinations. Glycerol-induced AKI, a representative model of heme protein-related renal injury, is characterized by oliguria, anuria, and a rapid decrease in renal excretory function. ^68^Ga-EDTA dynamic PET/CT images timely reflected the physiological change in the kidney, including the decrease of blood perfusion and urination. UUO is another typical renal fibrosis injury model. UUO initiates marked fibrosis and leads to the loss of nephrons. However, due to the powerful compensation by the contralateral kidney, whole renal excretion and GFR remains normal (32). However, a decline in renal plasma flow as well as renal excretion can be observed in the duration of obstruction on ^68^Ga-EDTA dynamic PET/CT images. ^68^Ga-EDTA-derived GFR and the enriched functional categories from renograms, including peak value, *T*_max_, BP, and EP, reflected the known functional characteristics of unilateral renal as well, which demonstrated the superiority of PET renal scanning in the measurement of split renal function. Furthermore, the bubble plot (Figure 7) comprehensively and simultaneously analyzed the split renal function as well as the overall situation of the examinees. We are however conscious that renal function should not be solely focused on the GFR value.

^99m^Tc-diethylenetriaminepentaacetic acid dynamic SPECT renal scanning is well-known for its unique advantages in assessing split kidney GFR and high sensitivity in detecting alterations in renal function. However, this method is far from meeting the needs of clinicians at present. Our study demonstrated the clear predominance of PET imaging in the visual assessment of organ function, especially renal excretion. The notable advantages of ^68^Ga-EDTA dynamic PET/CT over SPECT/CT lie in the significantly improved accuracy in providing both 3D renal information and GFR calculation. As a 2D imaging modality, SPECT is unable to collect renal depth information and leaves out a significant portion of radioactivity from 3D space, resulting in a less accurate measurement of GFR and misleading results for patients with severely injured kidneys or hyperfiltration (11). PET imaging allows researchers and clinicians to obtain quantitative renal excretory information (10, 25), which can be directly used for GFR measurement to understand glomerular filtration in a real-time and 3D fashion. Our previous report (16) and results from this study validated the feasibility of the AUC method for GFR calculation. We therefore consider that ^68^Ga-EDTA can be used as a novel renal glomerular filtration tracer, and ^68^Ga-EDTA dynamic renal PET can enable early and precise evaluation of renal function, which we suggest will overcome the shortcomings of SPECT imaging and transform the future landscape of the diagnosis of renal function.

Finally, some limitations should be noted. Our calculated GFR of the obstructed kidney in UUO mice (~0.05 ml/min) is overestimated owing to the existence of blood perfusion. Despite the known overestimation, our method demonstrated high feasibility and wide compatibility for mice with deformed kidneys and therefore may find better application in real-world practice. Further research is needed to establish a GFR calculation method to rule out the influence of the bladder and urine. Lee et al. (24) and Kirsting et al. (25) used the two-compartment pharmacokinetic modeling to measure murine and human GFR. The main point of this approach lies in the more accurate location of the extravascular functional renal cortex (EVRC), as well as the more precise quantification of the imaging data. However, for PET imaging of mice, the limited resolution of micro-PET made it difficult to precisely outline the EVRC, even with the help of semi-automated delineation. A higher resolution PET detector should be useful to improve detection efficiency.

## 5. Conclusions

This study comprehensively evaluated the performance of ^68^Ga-EDTA dynamic PET in the assessment of renal function. ^68^Ga-EDTA dynamic PET imaging helps clinicians and researchers visualize physiological changes in renal injury models, presents a clear predominance in precise split GFR measurements, and contributes to early warning of renal injury. ^68^Ga-EDTA dynamic PET is a meaningful method for evaluating renal function and is expected to have clinical applications in the coming years.

## Data availability statement

The original contributions presented in the study are included in the article/Supplementary material, further inquiries can be directed to the corresponding authors.

## Ethics statement

The animal study was reviewed and approved by the Institutional Animal Care and Use Committee of Tongji Medical College of Huazhong University of Science and Technology.

## Author contributions

YDi planned the study, acquired and complied the data, carried out the statistical analysis, and wrote the manuscript. YL, LZ, YDe, and HC acquired the data and processed the images. XL planned this study and reviewed this manuscript. DJ and WC proposed the original idea, planned the study, acquired and complied the data, and wrote and reviewed the manuscript. All authors contributed to the article and approved the submitted version.

## References

[1] Levey AS Eckardt KU Tsukamoto Y Levin A Coresh J Rossert J. Definition and classification of chronic kidney disease: a position statement from Kidney Disease: Improving Global Outcomes (KDIGO). Kidney Int. (2005) 67:2089–100. 10.1111/j.1523-1755.2005.00365.x 15882252

[2] Spanaus KS Kollerits B Ritz E Hersberger M Kronenberg F von Eckardstein A. Serum creatinine, cystatin C, and beta-trace protein in diagnostic staging and predicting progression of primary nondiabetic chronic kidney disease. Clin Chem. (2010) 56:740–9. 10.1373/clinchem.2009.138826 20224047

[3] Levey AS Coresh J Tighiouart H Greene T Inker LA. Measured and estimated glomerular filtration rate: current status and future directions. Nat Rev Nephrol. (2020) 16:51–64. 10.1038/s41581-019-0191-y 31527790

[4] Yuan X Tang W Shi W Yu L Zhang J Yuan Q. Determination of glomerular filtration rate (GFR) from fractional renal accumulation of iodinated contrast material: a convenient and rapid single-kidney CT-GFR technique. Eur Radiol. (2018) 28:2763–71. 10.1007/s00330-017-5289-7 29426992

[5] Ebrahimi B Textor SC Lerman LO. Renal relevant radiology: renal functional magnetic resonance imaging. Clin J Am Soc Nephrol. (2014) 9:395–405. 10.2215/CJN.02900313 24370767 PMC3913228

[6] Cheung CM Shurrab AE Buckley DL Hegarty J Middleton RJ Mamtora H. MR-derived renal morphology and renal function in patients with atherosclerotic renovascular disease. Kidney Int. (2006) 69:715–22. 10.1038/sj.ki.5000118 16395249

[7] Stevens LA Levey AS. Measured GFR as a confirmatory test for estimated GFR. J Am Soc Nephrol. (2009) 20:2305–13. 10.1681/ASN.2009020171 19833901

[8] Rudnick MR Leonberg-Yoo AK Litt HI Cohen RM Hilton S Reese PP. The controversy of contrast-induced nephropathy with intravenous contrast: what is the risk? Am J Kidney Dis. (2020) 75:105–13. 10.1053/j.ajkd.2019.05.022 31473019

[9] Ma YC Zuo L Zhang CL Wang M Wang RF Wang HY. Comparison of 99mTc-DTPA renal dynamic imaging with modified MDRD equation for glomerular filtration rate estimation in Chinese patients in different stages of chronic kidney disease. Nephrol Dial Transplant. (2007) 22:417–23. 10.1093/ndt/gfl603 17053082

[10] Werner RA Chen X Lapa C Koshino K Rowe SP Pomper MG. The next era of renal radionuclide imaging: novel PET radiotracers. Eur J Nuclear Med Mol Imaging. (2019) 46:1773–86. 10.1007/s00259-019-04359-8 31144061 PMC6647203

[11] Kang YK Park S Suh MS Byun SS Chae DW Lee WW. Quantitative single-photon emission computed tomography/computed tomography for glomerular filtration rate measurement. Nuclear Med Mol Imaging. (2017) 51:338–46. 10.1007/s13139-017-0491-8 29242728 PMC5721092

[12] James ML Gambhir SS. A molecular imaging primer: modalities, imaging agents, and applications. Physiol Rev. (2012) 92:897–965. 10.1152/physrev.00049.2010 22535898

[13] Aronson AL Ahrens FA. The mechanism of renal transport and excretion of ethylenediaminetetraacetate with interspecies comparisons. Toxicol Appl Pharmacol. (1971) 18:1–9. 10.1016/0041-008X(71)90309-7 5542825

[14] Hofman MS Hicks RJ. Gallium-68 EDTA PET/CT for renal imaging. Semin Nucl Med. (2016) 46:448–61. 10.1053/j.semnuclmed.2016.04.002 27553470

[15] Gündel D Pohle U Prell E Odparlik A Thews O. Assessing glomerular filtration in small animals using [(68)Ga]DTPA and [(68)Ga]EDTA with PET imaging. Mol Imag Biol. (2018) 20:457–64. 10.1007/s11307-017-1135-1 29063303

[16] Ding Y Zhang D Wang M Zhang L Liu Y Deng Y. Glomerular filtration rate calculation based on (68)Ga-EDTA dynamic renal PET. Am J Nuclear Med Mol Imaging. (2022) 12:54–62. 35535119 PMC9077168

[17] Breeman WA de Jong M de Blois E Bernard BF Konijnenberg M Krenning EP. Radiolabelling DOTA-peptides with 68Ga. Eur J Nuclear Med Mol Imaging. (2005) 32:478–85. 10.1007/s00259-004-1702-y 15655678

[18] Jiang D Ge Z Im HJ England CG Ni D Hou J. DNA origami nanostructures can exhibit preferential renal uptake and alleviate acute kidney injury. Nat Biomed Eng. (2018) 2:865–77. 10.1038/s41551-018-0317-8 30505626 PMC6258029

[19] Murase K Kitamura A Tachibana A Kusakabe Y Matsuura R Miyazaki S. Quantitative assessment of early experimental diabetes in rats using dynamic contrast-enhanced computed tomography. Eur J Radiol. (2010) 74:280–6. 10.1016/j.ejrad.2009.03.006 19346093

[20] Wesolowski MJ Conrad GR Šámal M Watson G Wanasundara SN Babyn P. A simple method for determining split renal function from dynamic (99m)Tc-MAG3 scintigraphic data. Eur J Nuclear Med Mol Imaging. (2016) 43:550–8. 10.1007/s00259-015-3216-1 26537286

[21] Hackstein N Wiegand C Langheinrich AC Rau WS. Measurement of glomerular filtration rate by low-dose iopromide plasma clearance. Acta Radiol. (2003) 44:162–5. 10.1034/j.1600-0455.2003.00037.x 12694102

[22] Gonzalez Melo M Fontana AO Viertl D Allenbach G Prior JO Rotman S. A knock-in rat model unravels acute and chronic renal toxicity in glutaric aciduria type I. Mol Genet Metab. (2021) 134:287–300. 10.1016/j.ymgme.2021.10.003 34799272

[23] Majd M Bar-Sever Z Santos AI De Palma D. The SNMMI and EANM procedural guidelines for diuresis renography in infants and children. J Nuclear Med. (2018) 59:1636–40. 10.2967/jnumed.118.215921 30275286 PMC6167528

[24] Lee HS Kang YK Lee H Han JH Moon BS Byun SS. Compartmental-modelling-based measurement of murine glomerular filtration rate using (18)F-fluoride PET/CT. Scie Rep. (2019) 9:11269. 10.1038/s41598-019-47728-x 31375734 PMC6677809

[25] Kersting D Sraieb M Seifert R Costa PF Kazek S Kessler L. First experiences with dynamic renal [(68)Ga]Ga-DOTA PET/CT: a comparison to renal scintigraphy and compartmental modelling to non-invasively estimate the glomerular filtration rate. Eur J Nuclear Med Mol Imaging. (2022) 49:3373–86. 10.1007/s00259-022-05781-1 35412053 PMC9002049

[26] Petejova N Martinek A. Acute kidney injury due to rhabdomyolysis and renal replacement therapy: a critical review. Crit Care. (2014) 18:224. 10.1186/cc13897 25043142 PMC4056317

[27] Werner RA Wakabayashi H Chen X Hirano M Shinaji T Lapa C. Functional renal imaging with 2-deoxy-2-(18)F-fluorosorbitol PET in rat models of renal disorders. J Nuclear Med. (2018) 59:828–32. 10.2967/jnumed.117.203828 29242399

[28] Hofman M Binns D Johnston V Siva S Thompson M Eu P. 68Ga-EDTA PET/CT imaging and plasma clearance for glomerular filtration rate quantification: comparison to conventional 51Cr-EDTA. J Nuclear Med. (2015) 56:405–9. 10.2967/jnumed.114.147843 25678493

[29] Shi S Zhang L Wu Z Zhang A Hong H Choi SR. [(68)Ga]Ga-HBED-CC-DiAsp: a new renal function imaging agent. Nuclear Med Biol. (2020) 82–83:17–24. 10.1016/j.nucmedbio.2019.12.005 31869735

[30] Galeotti L Ceccherini F Fucile C Marini V Di Paolo A Maximova N. Evaluation of pharmacokinetics and pharmacodynamics of deferasirox in pediatric patients. Pharmaceutics. (2021) 13:1238. 10.3390/pharmaceutics13081238 34452199 PMC8401444

[31] Qi Z Whitt I Mehta A Jin J Zhao M Harris RC. Serial determination of glomerular filtration rate in conscious mice using FITC-inulin clearance. Am J Physiol Renal Physiol. (2004) 286:F590–6. 10.1152/ajprenal.00324.2003 14600035

[32] Cochrane AL Kett MM Samuel CS Campanale NV Anderson WP Hume DA. Renal structural and functional repair in a mouse model of reversal of ureteral obstruction. J Am Soc Nephrol. (2005) 16:3623–30. 10.1681/ASN.2004090771 16221872

