# Supplementary material for: Quantitative assessment of renal functions using 68Ga-EDTA dynamic PET imaging in renal injury in mice of different origins
Source: Front Med (Lausanne). 2023 Mar 27;10:1143473. doi: 10.3389/fmed.2023.1143473 (PMC10083276; doi:10.3389/fmed.2023.1143473)
Supplement: Supplementary file 1 [file Data_Sheet_1.docx]

Supplementary Material

Quantitative assessment of Renal Functions via ^68^Ga-EDTA dynamic PET Imaging in kidney injury mice of varied origins

Ying Ding^1,2,3^, Yu Liu^1,2,3^, Li Zhang^1,2,3^, Yinqian Deng^1,2,3^, Huanyu Chen^1,2,3^, Xiaoli Lan^1,2,3^, Dawei Jiang^1,2,3*^, Wei Cao^1,2,3*^

^1^Department of Nuclear Medicine, Union Hospital, Tongji Medical college, Huazhong University of Science and Technology, Wuhan, China

^2^Hubei Key Laboratory of Molecular Imaging, Wuhan, China

^3^Key Laboratory of Biological Targeted Therapy, the Ministry of Education, Wuhan, China

*** Correspondence:**Wei Cao, M.D., Ph.D. Email: [caowei@hust.edu.cn](mailto:caowei@hust.edu.cn)

Dawei Jiang, Ph.D. Email: [daweijiang@hust.edu.cn](mailto:daweijiang@hust.edu.cn)

Keywords: Nuclear medicine, glomerular filtration rate, PET imaging, ^68^Ga-EDTA, kidney injury.

Running Title: PET imaging of kidney function

# Article types

Original Research. This article includes 5412 words, 7 figures, and 3 tables.

# Supplementary Figures


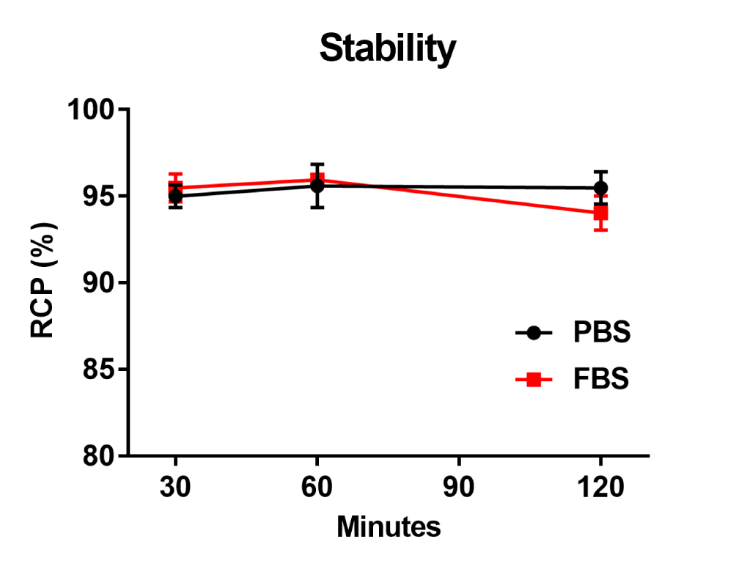


**Figure S1.** The stability of ^68^Ga-EDTA in PBS and FBS within 120 minutes


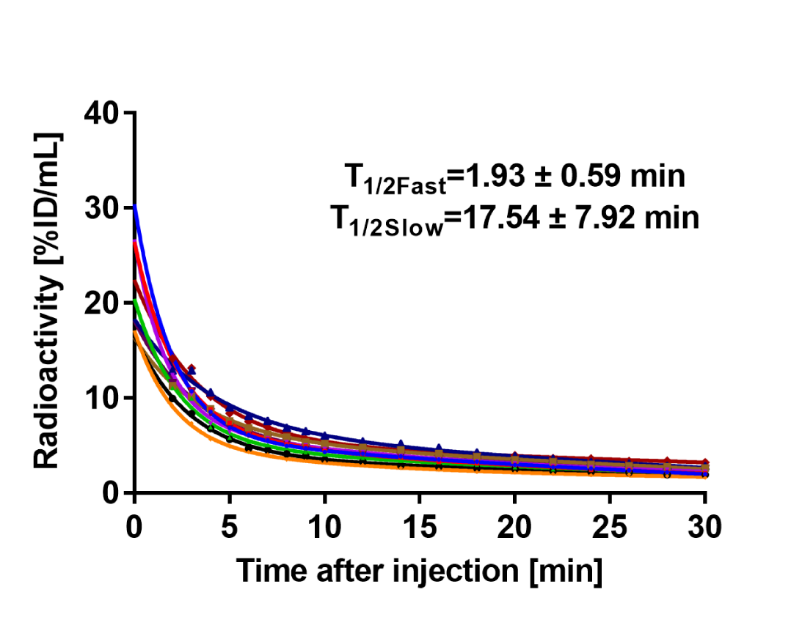


**Figure S2.** The washout curve of ^68^Ga-EDTA in healthy kidneys fitted by two-exponential decay


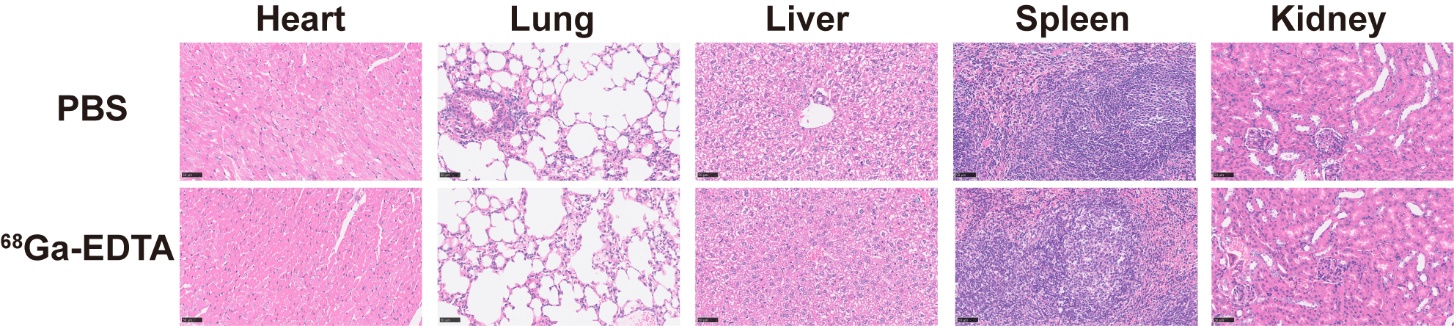


**Figure S3.** Histological sections in ^68^Ga-EDTA toxicity test (H & E staining, scale bar: 50 μm)

**
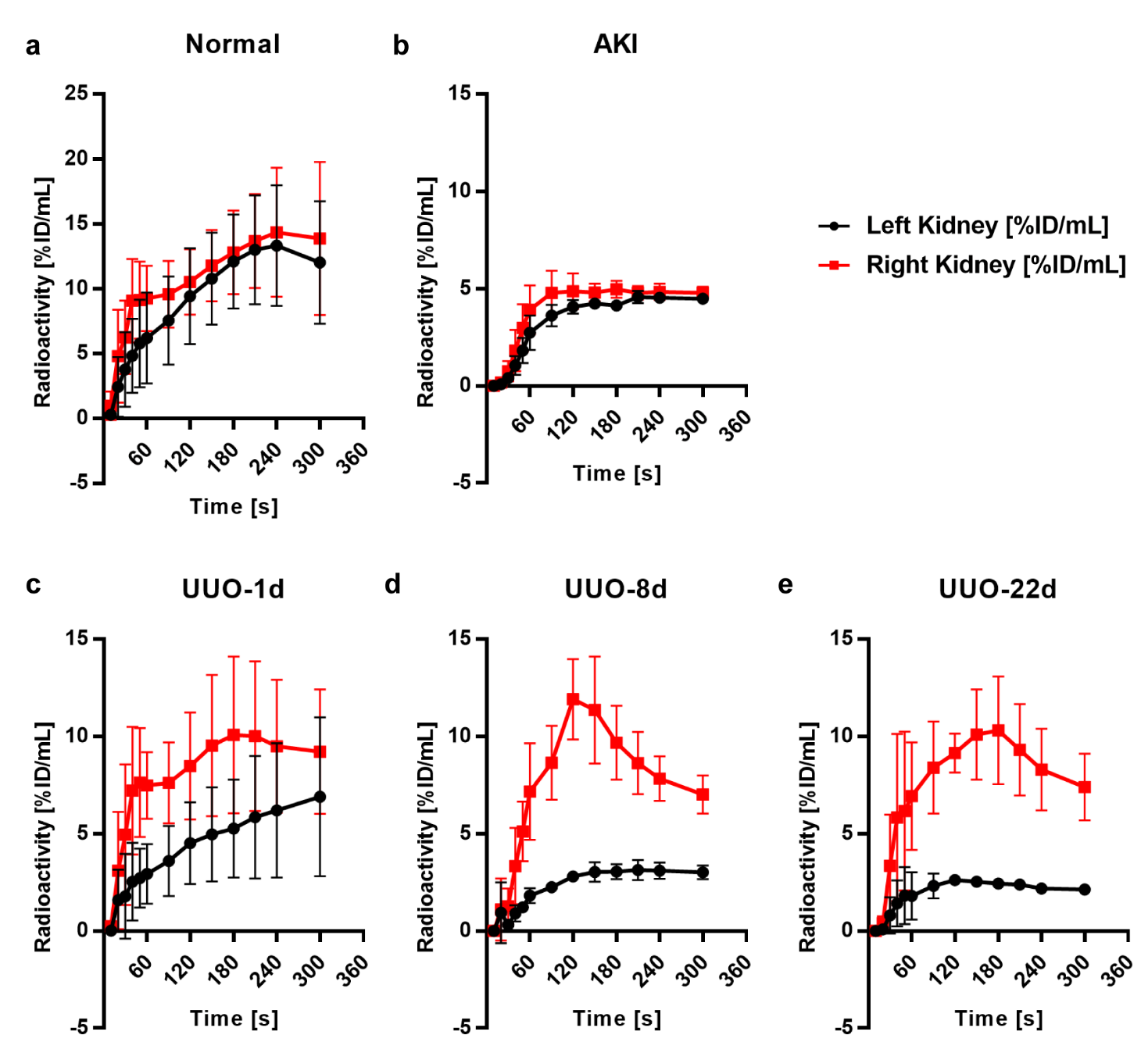
**

**Figure S4.** The ^68^Ga-EDTA blood perfusion curve for the split kidney


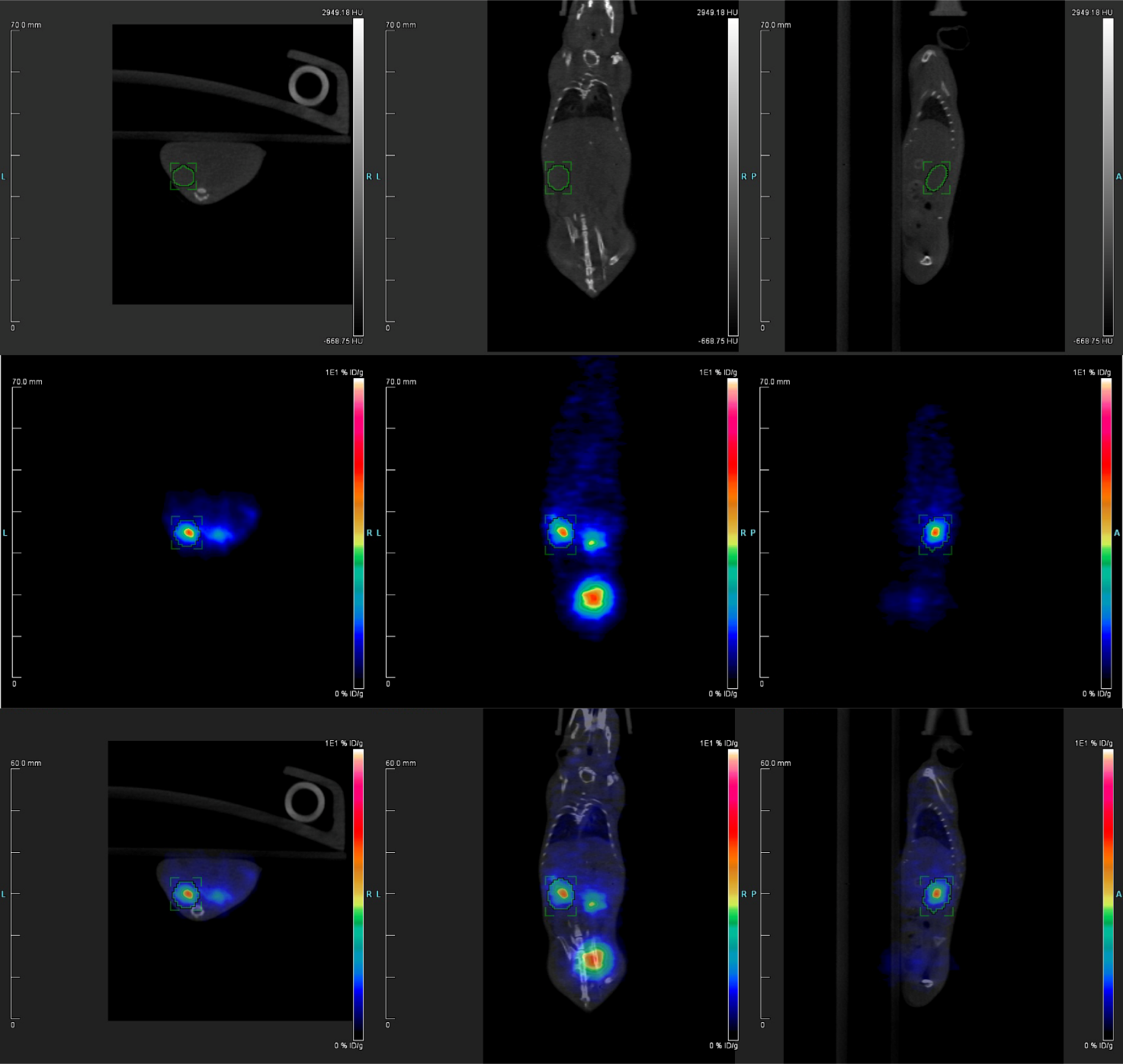


**Figure S5.** VOI drawing sample. The VOI was manually delineated first guided by the CT images, then adjusted by PET and PET/CT fusion images.

**Supplementary Tables**

Table S1. The blood biochemical parameters of the mice after the toxicity test of ^68^Ga-EDTA

|  | WBC  10^9^/L | RBC  10^12^/L | HGB  g/L | PLT  10^9^/L | ALT  U/L | AST  U/L | Cr  μmol/L |
| --- | --- | --- | --- | --- | --- | --- | --- |
| PBS | 4.4±2.7 | 6.7±0.8 | 104.0±11.1 | 568.7±77.5 | 60.3±4.6 | 168.9±54.5 | 21.9±4.0 |
| ^68^Ga-EDTA | 3.3±1.9 | 7.1±0.6 | 99.3±9.3 | 521.3±17.5 | 57.0±20.1 | 139.3±57.5 | 17.8±2.7 |
